# Neurodevelopmental status and adaptive behavior of pediatric patients with mucopolysaccharidosis II: a longitudinal observational study

**DOI:** 10.1186/s13023-023-02805-3

**Published:** 2023-11-16

**Authors:** Joseph Muenzer, Barbara K. Burton, Hernan M. Amartino, Paul R. Harmatz, Luis González Gutiérrez-Solana, Matilde Ruiz-Garcia, Yuna Wu, David Merberg, David Alexanderian, Simon A. Jones

**Affiliations:** 1 University of North Carolina at Chapel Hill, 101 Manning Drive CB# 7487, Medical School Wing E Room 117, Chapel Hill, NC 27599-7487 USA; 2 Ann & Robert H. Lurie Children’s Hospital of Chicago, Northwestern University, Chicago, IL USA; 3 Hospital Universitario Austral, Buenos Aires, Argentina; 4 UCSF Benioff Children’s Hospital Oakland, Oakland, CA USA; 5 Hospital Infantil Universitario Niño Jesús, Madrid, Spain; 6 National Institute of Pediatrics, Mexico City, Mexico; 7 Takeda Development Center Americas, Inc., Lexington, MA USA; 8 Takeda Development Center Americas, Inc., Cambridge, MA USA; 9 Merck, Boston, MA USA; 10 St Mary’s Hospital, Manchester University NHS Foundation Trust, University of Manchester, Manchester, UK

**Keywords:** Adaptive, Behavior, Cognitive, Decline, Function, Impairment, Mucopolysaccharidosis II, Neurodevelopment, Observational, Pediatric

## Abstract

**Background:**

Mucopolysaccharidosis (MPS) II is a rare, X-linked lysosomal storage disease. Approximately two-thirds of patients have central nervous system involvement with some demonstrating progressive cognitive impairment (neuronopathic disease). The natural history of cognitive and adaptive function in patients with MPS II is not well-defined. This 2-year, prospective, observational study evaluated the neurodevelopmental trajectories of boys with MPS II aged ≥ 2 years and < 18 years.

**Results:**

Overall, 55 patients were enrolled. At baseline, mean (standard deviation [SD]) age was 5.60 (3.32) years; all patients were receiving intravenous idursulfase. Cognitive and adaptive function were assessed using the Differential Ability Scales, Second Edition (DAS-II) General Conceptual Ability (GCA) and the Vineland Adaptive Behavior Scales, Second Edition (VABS-II) Adaptive Behavior Composite (ABC) scores, respectively. Baseline mean (SD) DAS-II GCA and VABS-II ABC scores were 78.4 (19.11) and 83.7 (14.22), respectively, indicating low cognitive function and moderately low adaptive behavior. Over 24 months, modest deteriorations in mean (SD) scores were observed for DAS-II GCA (−3.8 [12.7]) and VABS-II ABC (−2.0 [8.07]). Changes in DAS-II GCA scores varied considerably, and data suggested the existence of four potential patient subgroups: (1) patients with marked early impairment and rapid subsequent decline, (2) patients with marked early impairment then stabilization, (3) patients with mild early impairment then stabilization, and (4) patients without impairment who remained stable. Subgroup analyses revealed numerically greater DAS-II GCA score reductions from baseline in patients aged < 7 years at baseline (vs. those aged ≥ 7 years) and in patients with DAS-II GCA scores ≤ 70 at baseline (vs. those with scores > 70); between-group differences were nonsignificant. No clear subgroups or patterns were identified for individual changes in VABS-II ABC scores. In total, 49 patients (89.1%) reported ≥ 1 adverse event (AE) and nine patients (16.4%) reported serious AEs.

**Conclusions:**

Some patients with MPS II had rapid declines in cognitive ability, whereas others remained relatively stable after an initial decline. These insights provide a basis for more detailed analyses of different patient subgroups, which may enhance the definition and understanding of factors that influence cognitive and adaptive function in MPS II.

**Trial registration:**

ClinicalTrials.gov, NCT01822184. Registered retrospectively: April 2, 2013.

## Introduction

Mucopolysaccharidosis (MPS) II (Hunter syndrome; OMIM 309900) is a rare, X-linked, recessive lysosomal storage disease caused by pathogenic variants of the iduronate-2-sulfatase gene (*IDS*) [1]. In patients with MPS II, deficient activity of the enzyme iduronate-2-sulfatase (I2S) leads to lysosomal accumulation of glycosaminoglycans in organs and tissues throughout the body, resulting in damage to multiple organ systems [1]. The estimated worldwide incidence of MPS II is between 1 and 100,000 and 1 in 170,000 male live births [2, 3]. MPS II primarily affects male patients [1]; however, there have been reports of a small number of female patients [4–7].

MPS II is clinically heterogeneous in its presentation, with the severity of symptoms and rate of disease progression varying considerably between patients [1, 8, 9]. Common somatic clinical manifestations of the disease include coarse facial features, hepatosplenomegaly, hearing problems, severe airway obstruction, skeletal deformities, and cardiovascular disease [8, 9]. In addition to somatic manifestations, approximately two-thirds of patients have central nervous system (CNS) involvement, with some demonstrating progressive cognitive impairment and intense neurobehavioral symptoms (neuronopathic disease) [1, 9–12]. The natural history of cognitive and adaptive function in MPS II is not well-defined, but neurobehavioral symptoms include short attention span, high distractibility, impulsivity or heightened activity, sensation-seeking behavior, abnormal or inappropriate emotional and behavioral responses, lack of social skills, poor awareness of social cues, and poor sleep [12]. Patients with neuronopathic disease typically present with signs and symptoms of MPS II between the ages of 2 and 4 years and experience a developmental plateau in early childhood followed by deterioration of cognitive and adaptive behavioral functions [2, 8–10]. For these patients, death generally occurs in the second decade of life, whereas patients with non-neuronopathic disease may survive into their fifth or sixth decade [9, 13].

Intravenous (IV) enzyme replacement therapy (ERT) with recombinant I2S (idursulfase; Elaprase^®^, Takeda Pharmaceuticals USA, Inc., Lexington, MA, USA) is the current standard of care for patients with MPS II [14–18]. IV idursulfase is beneficial for many of the somatic manifestations of MPS II [14–17, 19]; however, IV idursulfase does not cross the blood–brain barrier at therapeutic concentrations, so it is not able to mitigate CNS manifestations [20, 21]. Thus, there remains an unmet need for a therapy that can treat the severe deterioration of cognitive and adaptive behavioral functions of many patients with MPS II. To this end, an intrathecal formulation of idursulfase (idursulfase-IT) has been developed with the aim of delivering the drug to the CNS [21]. Hematopoietic stem cell transplantation (HSCT) has demonstrated efficacy in patients with other MPS disorders, in particular MPS I; however, there is no clinical evidence from controlled trials to support its use in patients with MPS II [17]. Other therapies under investigation for the treatment of MPS II include gene therapy, which aims to provide the patient with a functional version of *IDS* [3, 22], and fusion-protein approaches, which use endogenous receptor-mediated transport mechanisms to deliver I2S to the CNS [23–25].

There is a need for more information on the natural history of neurodevelopmental status in patients with MPS II to aid disease management in these patients and to inform further development of therapies to treat neurological symptoms. The aim of this prospective, longitudinal, observational study was to evaluate the neurodevelopmental trajectories of a pediatric population with MPS II over 2 years.

## Methods and patients

### Study design

This 24-month, multicenter, multinational, prospective, longitudinal, observational study (NCT01822184) investigated the neurodevelopmental status of pediatric patients with MPS II using standardized instruments to assess cognitive and adaptive function. All recruitment and assessments were done within clinical hospital settings in Argentina, Mexico, Spain, the UK and the USA between January 2013 and October 2016. Enrollment was planned for up to 100 patients and was not based on statistical considerations.

**Fig. 1:**
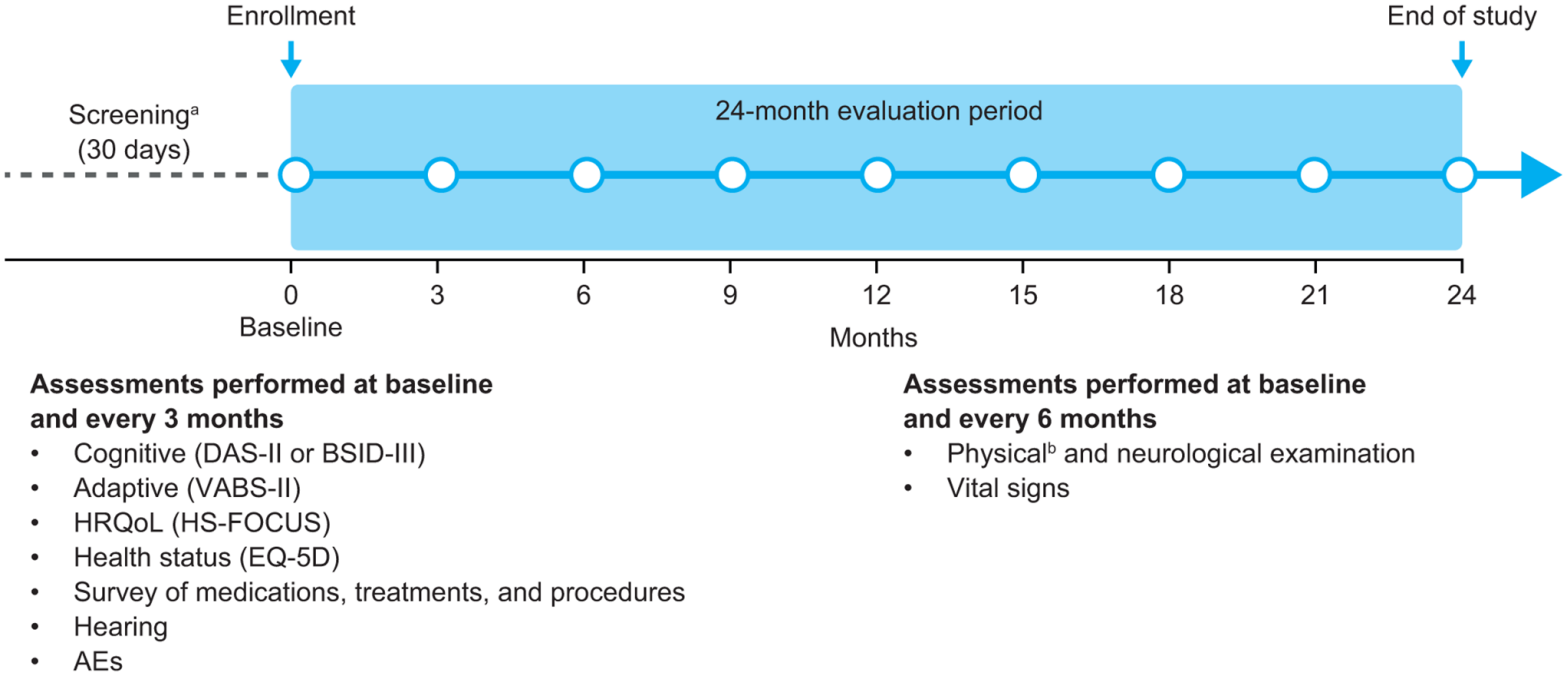
Study design. ^a^In addition to the assessments also performed at baseline, the screening period includes: obtaining/evaluating informed consent/assent; inclusion/exclusion criteria; genotyping; demographic information; medical history; and assessment of medications/therapies. ^b^Height and weight were measured at baseline and at months 12 and 24 only. AE, adverse event; BSID-III, Bayley Scales of Infant Development, Third Edition; DAS-II, Differential Ability Scales, Second Edition; HRQoL, health-related quality of life; HS-FOCUS, Hunter Syndrome Functional Outcomes for Clinical Understanding Scale; VABS-II, Vineland Adaptive Behavior Scales, Second Edition

Patients attended the study site for assessments at screening (day −30 to day −1), baseline (day 0) and then once every 3 months (± 14 days) until the month 24 (+ 14 days)/end-of-study visit (Fig. 1). For any patients who withdrew from the study or who were discontinued by a parent/caregiver or physician, assessments scheduled for month 24/end of study were completed in the 14 days after withdrawal/discontinuation. Neurodevelopmental testing was not repeated if it had been performed in the 8 weeks before the end-of-study visit.

Cognitive ability (Differential Ability Scales^®^ Second Edition [DAS-II]), adaptive behavior (Vineland Adaptive Behavior Scales^™^ Second Edition [VABS-II]), physical functioning and daily activities (Hunter Syndrome Functional Outcomes for Clinical Understanding Scale; HSFOCUS), health status (5-dimension Euro QoL questionnaire; EQ-5D), hearing, adverse events (AEs), and history of medication use, treatments, and procedures were assessed every 3 months. Physical and neurological examinations and vital sign checks were performed every 6 months.

### Patients

Boys aged ≥ 2 years and < 18 years with a confirmed diagnosis of MPS II and a DAS-II General Conceptual Ability (GCA) score ≥ 55 at screening were eligible for inclusion in the study. A diagnosis of MPS II was determined by a deficiency in I2S activity of ≤ 10% of the lower limit of normal as measured in plasma, fibroblasts, or leukocytes and either a documented *IDS* variant that left the fragile X-mental retardation 1 and 2 genes (*FMR1* and *FMR2*) intact, or a normal enzyme activity level of one other sulfatase as measured in plasma, fibroblasts, or leukocytes. Patients were required to have sufficient auditory capacity (with hearing aid[s], if needed) to complete the required assessments. Patients were eligible for inclusion regardless of MPS II treatment status. Patients who had participated in an interventional trial at or in the 30 days before enrollment were excluded. Patients were also excluded if they had clinically significant non–MPS II-related CNS involvement or medical or psychiatric comorbidities that could have affected administration and/or interpretation of protocol assessments, study data, and/or the integrity of the results [26].

During the period in which this observational trial took place, a phase 2/3 clinical trial of intrathecally administered idursulfase in patients with MPS II and early cognitive impairment was recruiting (HGT-HIT-094; NCT02055118)[27]. Certain inclusion criteria for the phase 2/3 trial may have influenced the results of the current study. At phase 2/3 trial screening, patients were required to have a recent history of cognitive decline; patients aged ≥ 3 and < 13 years were required to have either a DAS-II GCA score of 55–85, or a DAS-II GCA score of > 85 plus evidence of a ≥ 10-point decrease in this score over 12 months in the current study. Those aged ≥ 13 and < 18 years were required to have a baseline DAS-II GCA score of 55–85 at phase 2/3 trial screening plus evidence of a ≥ 10-point decrease in score over 12 months in the current study. Given that any patients who were enrolled into the phase 2/3 trial were subsequently discontinued from the current study, patients actively undergoing cognitive decline from the baseline values stated above may be underrepresented in the final study population.

### Study endpoints and assessments

The primary objective of this observational study was to evaluate the neurodevelopmental status of pediatric patients with MPS II over the course of 2 years. In this study, the DAS-II and the Survey Interview form of the VABS-II were used to assess cognitive function and adaptive behavior, respectively.

The DAS-II is a standardized tool used to measure cognitive function in children and adolescents with a wide range of cognitive abilities relative to a normative sample [26]. The test comprises a series of items administered by a qualified psychologist. The DAS-II is administered based on the child’s age and abilities and comprises two batteries: an early years test battery for children aged 2 years 6 months to 6 years 11 months (with a lower level for ages 2 years 6 months to 3 years 5 months and an upper level for ages 3 years 6 months to 6 years 11 months) and a school age test battery for those aged 7 years to 17 years 11 months. There is a 4-year normative overlap in the two batteries between the ages of 5 years through 8 years 11 months, since the early years battery is normed from age 2 years 6 months to 8 years 11 months, and the school age battery is normed from age 5 years through 17 years 11 months.

Outputs of the DAS-II include two standard composite scores (GCA and Special Nonverbal Composite [SNC]), three cluster scores (verbal, nonverbal, and spatial), and 10 component core subtest scores. Different sets of subtests are used in the various test batteries; six subtests are used in the upper-level early years and school age batteries, and four in the lower-level early years battery. The DAS-II GCA composite score is a measure of overall cognitive ability and has a normative mean of 100 and a standard deviation (SD) of 15, with higher values indicating better cognitive function.

The VABS-II is a standardized norm-referenced tool used to measure adaptive behavior in individuals from birth to age 90 years [28]. The VABS-II is administered by a qualified psychologist during a semi-structured interview with the patient’s parent or caregiver. It comprises one composite score (ABC), four domain scores (for communication, daily living skills, socialization, and motor skills), and 11 component subdomain scores. The VABS-II ABC composite score is an overall measure of adaptive behavioral ability and has a normative mean of 100 and a SD of 15, with higher scores indicating better adaptive behavior.

AE data were collected continuously during the study for all patients from the time of informed consent through month 24/end-of-study visit and/or until the event resolved or stabilized or an outcome was reached, whichever occurred first. All AEs were coded using the Medical Dictionary for Regulatory Activities version 16.1.

### Statistical methods

The primary analysis population consisted of all enrolled patients who had at least one baseline neurodevelopmental assessment. Data for all outcomes were summarized by descriptive statistics. Changes from baseline at month 12 and month 24 were analyzed for DAS-II GCA and SNC composite and cluster scores and VABS-II ABC scores and domain scores. Baseline was defined as day 0 or screening (if data for day 0 were missing). Changes from baseline at month 12 and month 24 were calculated only for patients with data available at both baseline and the time point assessed. Potential trends over time were evaluated by reviewing graphical plots.

A mixed-effects model for repeated measures (MMRM) was used to analyze the effects of baseline age (< 7 and ≥ 7 years [aligning with the DAS-II battery structure]) and DAS-II GCA score (≤ 70 and > 70) subgroups on changes from baseline in DAS-II GCA and VABS-II ABC scores. The MMRM contained categorical effects for visit, subgroup, and interaction. The model had an unstructured, within-patient covariance structure. Denominator degrees of freedom for tests of fixed effects were estimated using the Kenward–Roger approximation. Estimated least-squares mean change from baseline and standard errors (SEs) at each visit were plotted by baseline age and DAS-II GCA score subgroups, and least-squares mean difference (LSMD) in changes from baseline between the age and DAS-II GCA score subgroups were calculated. Exploratory statistical analyses were performed using the SAS software (SAS Institute, Inc., Cary, NC, USA) version 9.4.

## Results

### Study population

Of the 74 patients with MPS II screened, 55 were enrolled in this study. Three enrolled patients had DAS-II GCA scores that were miscalculated as ≥ 55 at baseline. Two participants had incomplete DAS-II GCA score assessments with true scores of 43 and 48, and one participant received a DAS-II GCA score of 53 and was evaluated for 24 months despite exclusion criteria. These miscalculations were identified after patient enrollment and study completion. Data for these participants were retained in the analysis according to the definition of the primary analysis population.

Baseline characteristics and patient disposition are summarized in Table 1. The mean (SD) age at baseline was 5.60 (3.32) years. All patients who enrolled in the study were receiving IV idursulfase at baseline. The most common *IDS* variant category was missense, and this variant type was present in 33 participants (60.0%). Twelve patients (21.8%) had null variants (‘frameshift’, ‘nonsense’, ‘premature truncation’, or ‘large deletion’). At baseline, 42 patients (76.4%) were aged < 7 years and 13 patients (23.6%) were aged ≥ 7 years. In total, 18 patients (32.7%) had a baseline DAS-II GCA score of ≤ 70 and 26 patients (47.3%) had a baseline DAS-II GCA score of > 70 (Table 1).

**Table 1:** Baseline characteristics and patient disposition

Characteristic/disposition	Study population (N = 55)
Male, n (%)	55 (100)
Age, years	
Mean (SD)	5.60 (3.316)
Median (min, max)	4.82 (2.0, 16.7)
Age subgroups, n (%)	
< 7 years	42 (76.4)
≥ 7 years	13 (23.6)
Race, n (%)	
Asian	4 (7.3)
Black or African American	1 (1.8)
White	47 (85.5)
Other	3 (5.5)
Height, cm	
Mean (SD)	111.58 (14.923)
Median (min, max)	110.10 (85.6, 152.0)
Weight, kg	
Mean (SD)	25.00 (10.514)
Median (min, max)	22.50 (14.6, 83.3)
Genotype category, n (%)	
Null variant (frameshift, nonsense, premature truncated, and large deletion)	12 (21.8)
Missense/presumed missense	33 (60.0)
Presumed splice site variant/splice site variant	10 (18.2)
Baseline DAS-II GCA score, n (%)	
≤ 70^a^	18 (32.7)
> 70	26 (47.3)
Missing	11 (20.0)
Receiving IV idursulfase, n (%)	
Yes	55 (100)
No	0 (0)
Patient disposition, n (%)	
Completed	23 (41.8)
Discontinued	32 (58.2)
Reasons for discontinuation	
Noncompliance with study procedure	1 (1.8)
Withdrawal^b^	4 (7.3)
Lost to follow-up^c^	2 (3.6)
Other, enrolled into phase 2/3 idursulfase-IT trial (HGT-HIT-094, NCT02055118)	25 (45.5)

^a^Two participants had incomplete DAS-II GCA score assessments, screening/baseline scores lower than 55 (true scores of 43 and 48). One participant received a DAS-II GCA score of 53 and was evaluated for 24 months despite exclusion criteria

^b^Of these patients, one withdrew because their parent/caregiver did not want to be contacted for end of study, one withdrew consent, one withdrew because the parent decided that they did not want their child to continue on the study, and one withdrew with no reason given

^c^One patient was lost to follow-up because it was not possible to contact their parents, and one patient missed three visits and was no longer responding to follow-up

DAS-II, Differential Ability Scales, Second Edition; GCA, General Conceptual Ability; idursulfase-IT, idursulfase administered intrathecally; IV, intravenous; SD, standard deviation

Overall, 23 patients (41.8%) completed the study. During the period in which this observational trial took place (first patient’s first visit: January 18, 2013), a phase 2/3 clinical trial of intrathecally administered idursulfase in patients with MPS II and early cognitive impairment was recruiting (HGT-HIT-094; NCT02055118)[27]. Of the 32 patients (58.2%) who did not complete this observational study, 25 were enrolled in the phase 2/3 trial of intrathecal idursulfase, four withdrew, two were lost to follow-up, and one was noncompliant with study procedures (Table 1). Assessment of the baseline characteristics of those patients who discontinued the study compared with those who completed it suggests that those who discontinued were typically younger, shorter, weighed less, had a lower proportion of patients with a confirmed baseline DAS-II GCA score > 70, and had a higher proportion of patients with null variants and with severe disease phenotypes (Table 2). Indeed, most patients who discontinued did so in the first year of the study (24/31 [77.4%]; one patient was missing discontinuation data); according to the last recorded study visit, the mean (SD) time on study of all those who discontinued was 9.0 (6.1) months (Table 3).

**Table 2:** Baseline characteristics and patient disposition, split according to study completion status

Characteristic/disposition	Completed study (n = 23)	Discontinued study (n = 32)
Male, n (%)	23 (100)	32 (100)
Age, years		
Mean (SD)	7.30 (3.694)	4.39 (2.411)
Median (min, max)	6.22 (3.4, 16.7)	3.49 (2.0, 12.4)
Age subgroups, n (%)		
< 7 years	15 (65.2)	27 (84.4)
≥ 7 years	8 (34.8)	5 (15.6)
Race, n (%)		
Asian	1 (4.3)	3 (9.4)
Black or African American	0 (0)	1 (3.1)
White	20 (87.0)	27 (84.4)
Other	2 (8.7)	1 (3.1)
Height, cm		
Mean (SD)	117.6 (13.7)	107.2 (14.4)
Median (min, max)	114.0 (101.0, 152.0)	104.1 (85.6, 143.4)
Weight, kg		
Mean (SD)	28.3 (14.0)	22.6 (6.3)
Median (min, max)	25.2 (16.0, 83.3)	21.2 (14.6, 37.5)
Genotype category, n (%)		
Null variant (frameshift, nonsense, premature truncation, and large deletion)	2 (8.7)	10 (31.3)
Missense/presumed missense	14 (60.9)	19 (59.4)
Splice site variant/presumed splice site variant	7 (30.4)	3 (9.4)
Phenotype category, n (%)		
Mild	9 (39.1)	3 (9.4)
Intermediate	4 (17.4)	2 (6.3)
Severe	7 (30.4)	25 (78.1)
Unclassifiable	3 (13.0)	2 (6.3)
Baseline DAS-II GCA score, n (%)		
≤ 70	7 (30.4)	11 (34.4)
> 70	14 (60.9)	12 (37.5)
Missing	2 (8.7)	9 (28.1)
Receiving IV idursulfase, n (%)		
Yes	23 (100)	32 (100)
No	0 (0)	0 (0)

DAS-II, Differential Ability Scales, Second Edition; GCA, General Conceptual Ability; IV, intravenous; SD, standard deviation

**Table 3:** Patient-level genotype, DAS-II scores, and study discontinuation data from the overall study population

		Study discontinuation				DAS-II GCA score		DAS-II nonverbal reasoning standard score^a^		DAS-II verbal standard score^a^		Variant data		
ID	Age (years)	Discontinuation (Y/N)	Study year (M)	Reason for discontinuation	Enrolled in 094 (Y/N)	Baseline (group)	EoS/ last visit	Baseline	EoS/ last visit	Baseline	EoS/ last visit	Type	Nucleotide change	Amino acid substitution
Patient A	3.1	Y	1 (M0)	Enrolled in 094 study	Y	66 (≤ 70)	N/A	71	N/A	64	N/A	Missense	c.1402C > T	p.R468W
Patient B	3.4	Y	1 (M0)	Enrolled in 094 study	Y	94 (> 70)	N/A	85	N/A	104	N/A	Large deletion	Deletion of exons 1–8	–
Patient C	10.1	Y	1 (M0)	Withdrew from study	N	79 (> 70)	N/A	84	N/A	86	N/A	Nonsense	c.36G > A	p.W12X
Patient D	7	Y	1 (M0)	Enrolled in 094 study	Y	65 (≤ 70)	N/A	74	N/A	63	N/A	Nonsense	c.1186C > T	p.Q396X
Patient E	4.4	Y	1 (M0)	Enrolled in 094 study	Y	43 (≤ 70)	N/A	78	N/A	59	N/A	Missense	c.686A > G	p.H229R
Patient F	3.4	Y	1 (M3)	Enrolled in 094 study	Y	83 (> 70)	66	92	74	76	86	Missense	c.1472C > T	p.S491F
Patient G	2.4	Y	1 (M3)	Enrolled in 094 study	Y	Missing	83	Missing	83	Missing	87	Missense	c.685C > T	p.H229Y
Patient H	4.1	Y	1 (M3)	Enrolled in 094 study	Y	63 (≤ 70)	75	96	83	85	94	Complete deletion/large rearrangement	–	–
Patient I	12.4	Y	1 (M6)	Noncompliance	N	91 (> 70)	77	92	80	92	89	Intronic	c.1181-1G > C	–
Patient J	3.9	Y	1 (M6)	Enrolled in 094 study	Y	64 (≤ 70)	64	75	87	79	69	Nonsense	c.1093G > T	p.G365X
Patient K	5.2	Y	1 (M6)	Withdrew from study	N	Missing	47	68	63	69	65	Missense	c.998C > T	p.S333L
Patient L	2.2	Y	1 (M6)	Enrolled in 094 study	Y	Missing	78	Missing	86	Missing	72	Missense	c.1403G > C	p.R468P
Patient M	2.3	Y	1 (M12)	Enrolled in 094 study	Y	Missing	104	Missing	107	Missing	100	Frameshift	c.993delT	p.F331fsX8
Patient N	6.5	Y	1 (M9)	Enrolled in 094 study	Y	60 (≤ 70)	67	79	100	85	87	Missense	c.134A > G	p.D45G
Patient O	3.7	Y	1 (M9)	Enrolled in 094 study	Y	68 (≤ 70)	63	88	86	68	57	Splice site	c.1122C > T	p.G374G
Patient P	4.9	Y	1 (M9)	Lost to follow-up	N	59 (≤ 70)	37	70	55	63	39	Missense	c.1025A > C	p.H342P
Patient Q	3.5	Y	1 (M9)	Enrolled in 094 study	Y	Missing	55	Missing	84	Missing	54	Missense	c.1400C > T	p.P467L
Patient R	2.3	Y	1 (M9)	Enrolled in 094 study	Y	Missing	86	Missing	81	Missing	94	Missense	c.283A > G	p.R95G
Patient S	3	Y	1 (M9)	Enrolled in 094 study	Y	82 (> 70)	52	88	71	79	90	Unclassifiable^b^	c.785delC^b^	–
Patient T	8.1	Y	1 (M9)	Withdrew from study	N	55 (≤ 70)	71	74	79	45	77	Missense	c.1403G > A	p.R468Q
Patient U	3.3	Y	1 (M12)	Enrolled in 094 study	Y	87 (> 70)	77	84	105	93	92	Missense	c.1403G > A	p.R468Q
Patient V	2.9	Y	1 (M12)	Enrolled in 094 study	Y	95 (> 70)	74	85	71	105	108	Frameshift	c.542insA	p.N181fsX17
Patient W	5.1	Y	1 (M12)	Enrolled in 094 study	Y	56 (≤ 70)	45	77	64	70	57	Missense	c.1504T > G	p.W502G
Patient X	3.3	Y	1 (M12)	Screened for 094 study	N	90 (> 70)	88	90	102	90	93	Missense	c.1504T > G	p.W502G
Patient Y	4	Y	2 (M15)	Enrolled in 094 study	Y	96 (> 70)	104	98	118	114	117	Missense	c.134A > G	p.D45G
Patient Z	3.4	Y	2 (M15)	Enrolled in 094 study	Y	89 (> 70)	80	87	116	92	79	Intronic	c.709-2A > G	–
Patient AA	2.9	Y	2 (M15)	Enrolled in 094 study	Y	92 (> 70)	79	91	89	93	96	Missense	c.1402C > T	p.R468W
Patient AB	7.3	Y	2 (M15)	Lost to follow-up	N	122 (> 70)	128	112	112	101	136	Missense	c.425C > T	p.S142F
Patient AC	2.1	Y	2 (M21)	Enrolled in 094 study	Y	Missing	61	Missing	79	Missing	60	Frameshift	c.1091delC	p.P364fsX26
Patient AD	5.7	Y	2 (M21)	Screened for 094 study	N	55 (≤ 70)	56	69	64	54	55	Missense	c.262C > T	p.R88C
Patient AE	2	Y	2 (M21)	Withdrew from study	Y	Missing	87	Missing	88	Missing	96	Missense	c.998C > T	p.S333L
Patient AF	2.4	Y	Missing	Enrolled in 094 study	Y	Missing	Missing	Missing	Missing	Missing	Missing	Frameshift	c.1381_1384 dupATTG	p.A462fsX3
Patient AG	5.5	N	N/A	N/A	N	88 (> 70)	111	112	140	98	93	Missense	c.1400C > G	p.P467R
Patient AH	4	N	N/A	N/A	N	110 (> 70)	104	121	116	104	100	Missense	c.1568A > G	p.Y523C
Patient AI	4.8	N	N/A	N/A	N	113 (> 70)	102	115	95	106	99	Missense	c.1406C > T	p.P469L
Patient AJ	4.9	N	N/A	N/A	N	59 (≤ 70)	33	73	32	73	45	Missense	c.257C > T	p.P86L
Patient AK	3.7	N	N/A	N/A	N	70 (≤ 70)	34	81	47	85	36	Missense	c.257C > T	p.P86L
Patient AL	8.9	N	N/A	N/A	N	81 (> 70)	87	90	89	80	86	Missense	c.329G > A	p.R110K
Patient AM	6.2	N	N/A	N/A	N	62 (≤ 70)	66	77	85	71	75	Intronic	c.879 + 1G > T	–
Patient AN	6.2	N	N/A	N/A	N	102 (> 70)	108	87	91	99	122	Splice site	c.1122C > T	p.G374G
Patient AO	5.8	N	N/A	N/A	Y	90 (> 70)	88	93	88	96	87	Missense	c.239A > G	p.Q80R
Patient AP	12.8	N	N/A	N/A	N	Missing	92	Missing	94	83	81	Splice site	c.1122C > T	p.G374G
Patient AQ	15.4	N	N/A	N/A	N	98 (> 70)	94	94	103	89	103	Missense	c.281G > A	p.G94D
Patient AR	16.7	N	N/A	N/A	N	79 (> 70)	82	82	90	81	87	Splice site	c.1122C > T	p.G374G
Patient AS	7.9	N	N/A	N/A	N	81 (> 70)	74	85	86	77	65	Missense	c.1387T > C	p.Y463H
Patient AT	4.3	N	N/A	N/A	N	Missing	38	Missing	62	Missing	31	Missense	c.283A > G	p.R95G
Patient AU	6.4	N	N/A	N/A	N	104 (> 70)	105	110	107	71	93	Missense	c.253G > A	p.A85T
Patient AV	3.4	N	N/A	N/A	N	105 (> 70)	118	105	121	104	122	Missense	c.824A > T	p.D275V
Patient AW	4	N	N/A	N/A	N	48 (≤ 70)	37	61	49	69	41	Intronic	c.708 + 1G > A	–
Patient AX	7	N	N/A	N/A	N	86 (> 70)	78	78	78	83	73	Splice site	c.1122C > T	p.G374G
Patient AY	5.4	N	N/A	N/A	N	53 (≤ 70)	40	79	54	50	36	Missense	c.998C > T	p.S333L
Patient AZ	5.4	N	N/A	N/A	N	75 (> 70)	74	103	80	69	43	Intronic	c.1181-1G > A	–
Patient BA	7.9	N	N/A	N/A	N	72 (> 70)	71	96	93	46	38	Missense	c.592G > A	p.D198N
Patient BB	12.1	N	N/A	N/A	N	56 (≤ 70)	52	60	53	47	38	Nonsense	c.1327C > T	p.R443X
Patient BC	9	N	N/A	N/A	N	65 (≤ 70)	66	61	69	73	63	Nonsense	c.1327C > T	p.R443X

^a^The DAS-II nonverbal and verbal scores are for the early years or school age battery, as appropriate for the age of the patient in each case

^b^The genotype description reported for patient S was inconsistent with reference sequence. The variant recorded by the study site is a deletion of cytosine at position 785, but the reference nucleic acid at that position should be a thymine. As such, the variant for this patient was listed as ‘unclassifiable’

DAS-II, Differential Ability Scales, Second Edition; EoS, end of study; GCA, General Conceptual Ability; ID, anonymized patient identifier; M, month; N/A, not applicable

### DAS-II standard scores in the overall study population

Mean (SD) DAS-II composite and cluster scores at baseline and at months 12 and 24 are shown in Table 4. The baseline mean (SD) DAS-II GCA score for the overall study population (n = 44) was 78.4 (19.11), indicating low cognitive function (in the 2–8 percentile). Cognitive function at baseline varied widely between patients, with individual DAS-II GCA scores at baseline ranging from 43 to 122, consistent with the wide range of cognitive ability seen in MPS II. Over 24 months, there were modest mean changes from baseline in the DAS-II GCA score and its component cluster scores. At month 24, the mean (SD) changes from baseline ranged from −3.8 (12.71; n = 20) for the DAS-II GCA score to −6.4 (17.66; n = 21) for the verbal cluster score (Table 4).

**Table 4:** DAS-II standard scores and changes from baseline in the overall study population

	DAS-II standard scores (n = 54)				
	Cluster			Composite	
	Verbal	Nonverbal	Spatial	GCA	SNC
Baseline					
n	46	45	35	44	34
Mean (SD)	80.1 (17.46)	86.0 (14.52)	77.6 (20.56)	78.4 (19.11)	78.8 (18.23)
Change from baseline at month 12					
n	28	27	22	27	21
Mean (SD)	−1.1 (11.67)	4.0 (12.97)	−2.5 (13.98)	−0.9 (9.39)	1.2 (11.18)
Change from baseline at month 24					
n	21	20	20	20	19
Mean (SD)	−6.4 (17.66)	−5.3 (16.81)	−3.9 (18.19)	−3.8 (12.71)	−5.4 (16.43)

Changes from baseline were calculated using data only from patients with data available at both baseline and month 12 or at both baseline and month 24

For DAS-II cluster and composite scores: mean = 100; standard scores of ≤ 69, 70–79, 80–89, and 90–109 indicate very low, low, below average, and average function, respectively

DAS-II, Differential Ability Scales, Second Edition; GCA, General Conceptual Ability; SD, standard deviation; SNC, special nonverbal composite

**Fig. 2:**
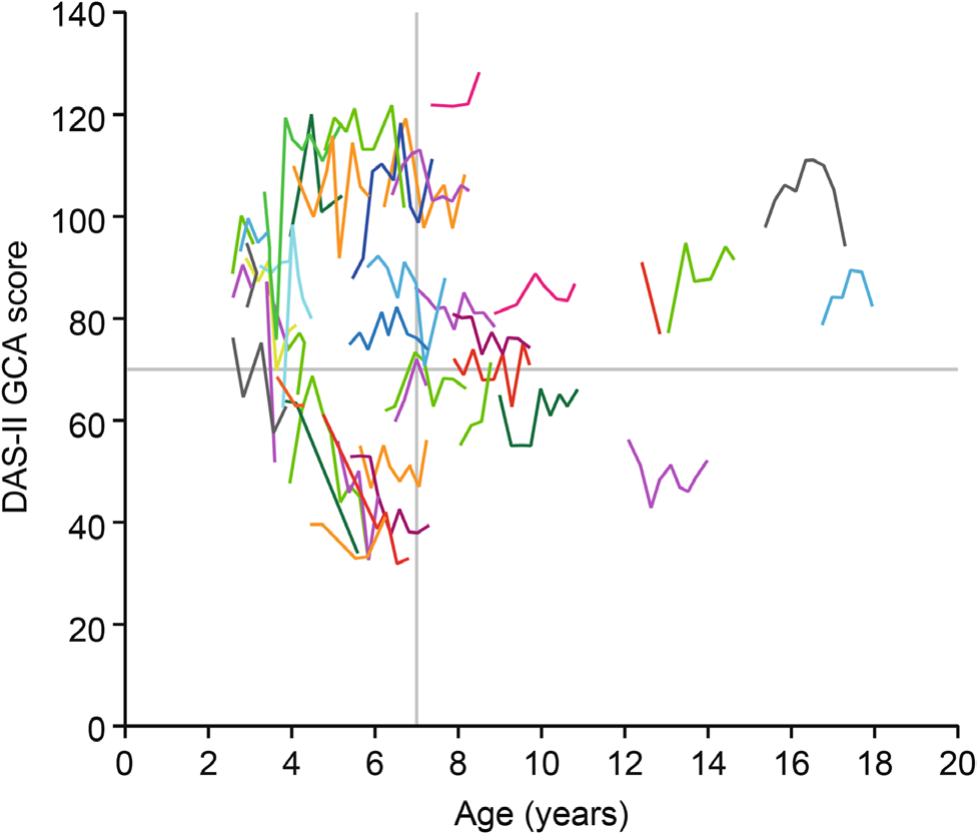
Individual DAS-II GCA scores^a^ for the overall study population by chronological age. ^a^The horizontal and vertical lines show the upper limits of the subgroup analyses for baseline DAS-II GCA score and age, respectively. The only subgroup that showed a clear decline in cognitive function were younger patients (aged < 7 years) who had a lower DAS-II GCA score at baseline (≤ 70), as depicted in the left-hand lower quadrant. Decline in cognitive function decline was less clear in the other baseline age/DAS-II GCA score subgroups, which may have been due to patients having non-neuronopathic disease (and therefore not experiencing a decline in cognitive function) or neuronopathic disease that was in the plateau stage of cognitive impairment. DAS-II, Differential Ability Scales, Second Edition; GCA, General Conceptual Ability

Changes in DAS-II GCA scores over time in individual patients varied widely. Although some patients experienced rapid declines in cognitive ability, others remained stable over the 24-month observational period (Fig. 2). Four potential subgroups were discernable based on a visual assessment of the DAS-II GCA score plots: (1) patients who had evidence of early (i.e. onset at a young age) cognitive impairment and experienced a rapid decline in cognitive function during the study; (2) patients who experienced marked early cognitive impairment before study entry, but remained in a plateau phase during the study; (3) patients who had mild early cognitive impairment at baseline and who remained stable throughout the study; and (4) patients with non-neuronopathic disease whose scores suggested no cognitive impairment at baseline (based on the interpretation of the attending physician) and did not experience cognitive function decline during the study.

### DAS-II GCA scores by baseline age and DAS-II GCA score subgroups

Changes from baseline in mean (SD) DAS-II GCA score by baseline age and DAS-II GCA score subgroups are shown in Table 5. Cognitive function at baseline was similar in the subgroup of patients aged < 7 years (mean [SD] DAS-II GCA score, early years battery: 78.3 [19.50]; n = 32) and in that of patients ≥ 7 years (mean DAS-II GCA score, school age battery: 78.7 [18.85]; n = 12). At month 12, mean (SD) DAS-II GCA score decreased from baseline in the subgroup of patients < 7 years by −2.3 (10.07; n = 19), compared with a mean (SD) increase from baseline in DAS-II GCA scores of 2.4 (7.03; n = 8) in the subgroup of patients aged ≥ 7 years. At month 24, a greater mean (SD) decline in DAS-II GCA score from baseline was observed in the subgroup aged < 7 years (−4.8 [14.98]; n = 14) than in the subgroup aged ≥ 7 years (−1.5 [4.59]; n = 6). There were no significant differences in the LSMD (SE) in DAS-II GCA scores from baseline between the two age subgroups at month 12 (−7.1 [3.97]; *P* = 0.0852) and month 24 (−8.9 [5.57]; *P* = 0.1239). Six patients switched from the early years battery to the school age battery, but their ages at the time of the assessments remained within the co-normed range of the tests.

**Table 5:** Change from baseline in DAS-II GCA scores by baseline age and DAS-II GCA score subgroups

	Patient subgroup			
	Baseline age < 7 years (n = 42)	Baseline age ≥ 7 years (n = 13)	Baseline DAS-II GCA score ≤ 70 (n = 18)	Baseline DAS-II GCA score > 70 (n = 26)
Baseline				
n	32	12	18	26
Mean (SD)	78.3 (19.50)	78.7 (18.85)	59.3 (7.01)	91.7 (12.14)
Change from baseline at month 12				
n	19	8	7	20
Mean (SD)	−2.3 (10.07)	2.4 (7.03)	−2.4 (9.83)	−0.4 (9.44)
Change from baseline at month 24				
n	14	6	7	13
Mean (SD)	−4.8 (14.98)	−1.5 (4.59)	−12.1 (14.51)	0.7 (9.38)

Changes from baseline were calculated using data only from patients with data available at both baseline and month 12 or at both baseline and month 24

For DAS-II cluster and composite scores: mean = 100; standard scores of ≤ 69, 70–79, 80–89, and 90–109 indicate very low, low, below average, and average function, respectively

DAS-II, Differential Ability Scales, Second Edition; GCA, General Conceptual Ability; SD, standard deviation

**Fig. 3:**
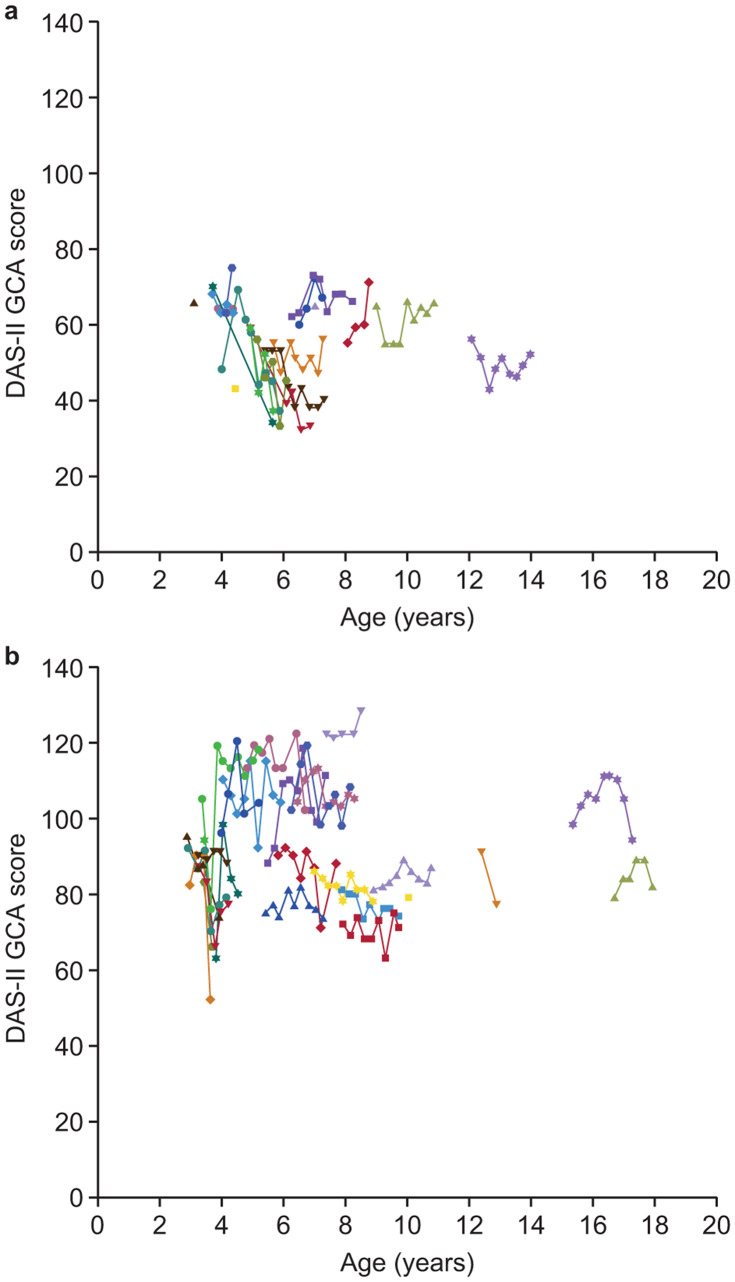
Individual DAS-II GCA scores by baseline DAS-II GCA score **(a)** ≤ 70 and **(b)** > 70. DAS-II, Differential Ability Scales, Second Edition; GCA, General Conceptual Ability

For the two DAS-II GCA score subgroups (baseline DAS-II GCA scores of ≤ 70 [very low cognitive ability] and > 70 [low cognitive ability or better]), the mean (SD) baseline DAS-II GCA scores were 59.3 (7.01; n = 18) and 91.7 (12.14; n = 26), respectively. Over the course of the 24-month observation period, patients with a baseline DAS-II GCA score ≤ 70 had a trend towards cognitive decline (Fig. 3a), whereas cognitive function in patients with a baseline DAS-II GCA score > 70 remained stable (Fig. 3b). Mean (SD) changes in DAS-II GCA score from baseline at month 12 were −2.4 (9.83; n = 7) in the ≤ 70 score subgroup and −0.4 (9.44; n = 20) in the > 70 score subgroup; at month 24, changes from baseline were −12.1 (14.51; n = 7) and 0.7 (9.38; n = 13), respectively. LSMD (SE) between changes in DAS-II GCA scores from baseline in the two DAS-II GCA score subgroups were −2.4 (4.09; *P* = 0.5657) at month 12 and −7.4 (4.95; *P* = 0.1461) at month 24.

### VABS-II standard scores in the overall study population

**Fig. 4:**
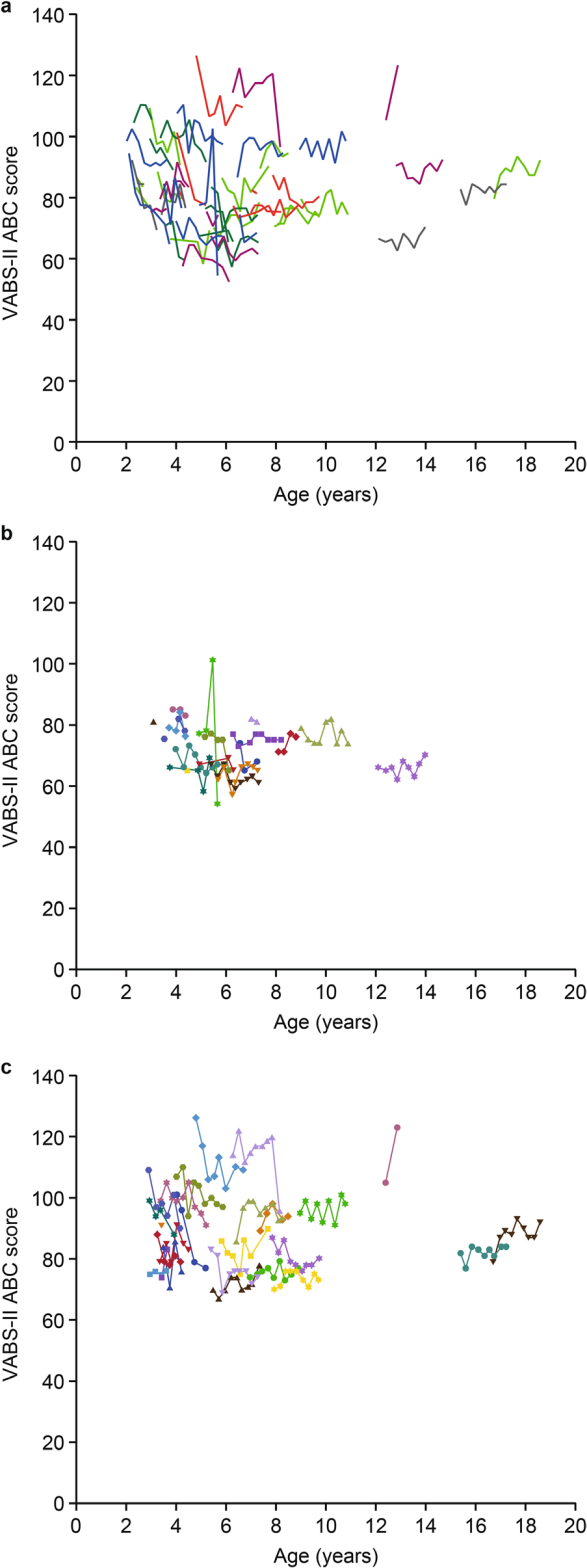
Individual VABS-II ABC scores **(a)** overall and by baseline DAS-II GCA scores **(b)** ≤ 70 and **(c)** > 70. ABC, Adaptive Behavior Composite; DAS-II, Differential Ability Scales, Second Edition; GCA, General Conceptual Ability; VABS-II, Vineland Adaptive Behavior Scales, Second Edition

**Fig. 5:**
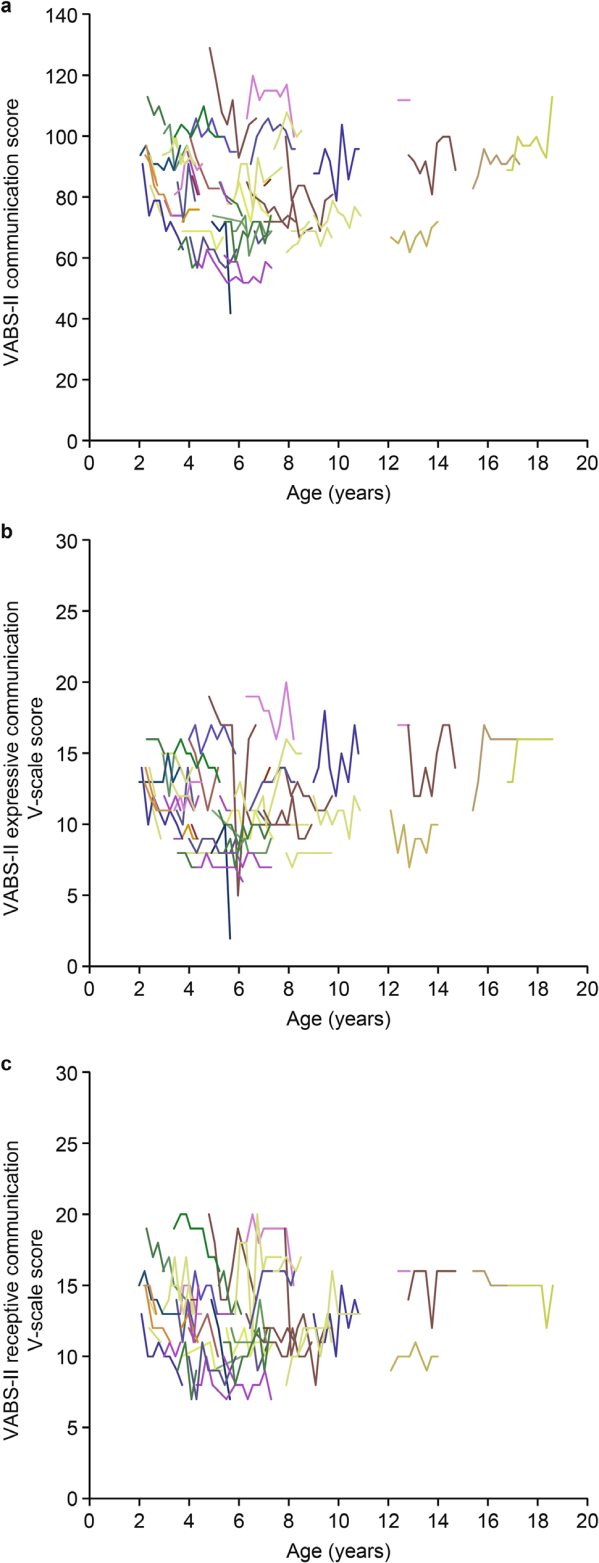
Individual VABS-II **(a)** communication domain standard scores and V-scale scores for **(b)** expressive and **(c)** receptive communication. VABS-II, Vineland Adaptive Behavior Scales, Second Edition

Mean (SD) VABS-II ABC and domain scores at baseline and at months 12 and 24 are shown in Table 6. The baseline mean (SD) VABS-II ABC score was 83.7 (14.22) for the overall study population (n = 53), indicating moderately low adaptive behavior (3–17 percentile range). At month 24, there was a modest mean (SD) change from baseline in VABS-II ABC score of −2.0 (8.07; n = 21). As with DAS-II GCA scores, changes in VABS-II ABC scores in individual patients varied widely (changes from baseline at month 24 ranged from −18 to 13; Fig. 4a). Mean (SD) changes from baseline in VABS-II domain scores at month 24 were −1.3 (10.23; n = 21) for the communication domain, −4.8 (10.32; n = 21) for the daily living skills domain, −1.7 (11.18; n = 21) for the socialization domain, and −0.1 (10.14; n = 21) for the motor skills domain. Individual changes in the VABS-II communication domain standard scores and the V-scale scores for expressive communication and receptive communication are shown in Fig. 5.

**Table 6:** VABS-II standard scores and changes from baseline in the overall study population

	VABS-II standard scores (n = 54)				
	ABC	Communication	Daily living skills	Socialization	Motor skills
Baseline					
n	53	53	53	53	53
Mean (SD)	83.7 (14.22)	85.0 (14.77)	87.1 (15.05)	86.9 (14.72)	84.4 (16.42)
Change from baseline at month 12					
n	29	30	30	30	29
Mean (SD)	−2.4 (7.64)	−2.9 (10.11)	−1.8 (9.78)	−0.2 (8.41)	−4.3 (12.82)
Change from baseline at month 24					
n	21	21	21	21	21
Mean (SD)	−2.0 (8.07)	−1.3 (10.23)	−4.8 (10.32)	−1.7 (11.18)	−0.1 (10.14)

Changes from baseline were calculated using data only from patients with data available at both baseline and month 12 or at both baseline and month 24

For VABS-II: mean = 100; standard scores of ≤ 70, 71–85, and 86–114 indicate a low, moderately low, and adequate adaptive level, respectively

ABC, Adaptive Behavior Composite; SD, standard deviation; VABS-II, Vineland Adaptive Behavior Scales, Second Edition

### VABS-II ABC scores by baseline age and DAS-II GCA score subgroups

Changes from baseline in mean (SD) VABS-II ABC score by baseline age and DAS-II GCA score subgroups are shown in Table 7. Adaptive behavior was similar in both age subgroups at baseline. The mean (SD) change from baseline in VABS-II ABC score at month 12 was −4.0 (7.64; n = 20) in the subgroup aged < 7 years and 1.0 (6.84; n = 9) in the subgroup aged ≥ 7 years. At month 24, the mean (SD) change in VABS-II ABC score from baseline was −4.4 (8.42; n = 13) in the subgroup aged < 7 years and 1.9 (6.06; n = 8) in the subgroup aged ≥ 7 years. LSMD (SE) in the changes in VABS-II ABC scores from baseline between the two age subgroups were −7.0 (2.98; *P* = 0.0207) at month 12 and −8.3 (3.44; *P* = 0.0175) at month 24.

**Table 7:** Change from baseline in VABS-II ABC scores by baseline age and DAS-II GCA score subgroups

	Patient subgroup			
	Baseline age < 7 years (n = 42)	Baseline age ≥ 7 years (n = 13)	Baseline DAS-II GCA score ≤ 70 (n = 18)	Baseline DAS-II GCA score > 70 (n = 26)
Baseline				
n	41	12	18	24
Mean (SD)	83.9 (15.12)	82.9 (11.14)	73.8 (7.00)	90.3 (14.73)
Change from baseline at month 12				
n	20	9	7	19
Mean (SD)	−4.0 (7.64)	1.0 (6.84)	−2.4 (5.83)	−0.9 (7.30)
Change from baseline at month 24				
n	13	8	5	14
Mean (SD)	−4.4 (8.42)	1.9 (6.06)	−2.8 (4.09)	−1.8 (9.63)

Changes from baseline were calculated using data only from patients with data available at both baseline and month 12 or at both baseline and month 24

For VABS-II: mean = 100; standard scores of ≤ 70, 71–85, and 86–114 indicate a low, moderately low, and adequate adaptive level, respectively

ABC, Adaptive Behavior Composite; DAS-II, Differential Ability Scales, Second Edition; GCA, General Conceptual Ability; SD, standard deviation; VABS-II, Vineland Adaptive Behavior Scales, Second Edition

There was a large difference in mean (SD) VABS-II ABC scores at baseline between the subgroup with baseline DAS-II GCA scores ≤ 70 (73.8 [7.00]; n = 18) and the subgroup with scores > 70 (90.3 [14.73]; n = 24). The mean (SD) change from baseline in VABS-II ABC score at month 12 was −2.4 (5.83; n = 7) in the ≤ 70 score subgroup and −0.9 (7.30; n = 19) in the > 70 score subgroup. At month 24, the mean (SD) change from baseline in VABS-II ABC score was −2.8 (4.09; n = 5) in the ≤ 70 score subgroup and −1.8 (9.63; n = 14) in the > 70 score subgroup. LSMD (SE) between changes in VABS-II ABC scores from baseline for patients with baseline DAS-II GCA scores of ≤ 70 or > 70 were −1.9 (3.16; *P* = 0.5545) at month 12 and 0.3 (3.92; *P* = 0.9484) at month 24.

No clear subgroups or patterns were identifiable in the individual patient plots of VABS-II ABC score by baseline DAS-II GCA subgroup (Fig. 4b and c).

### Adverse events

In total, 49 patients (89.1%) had at least one AE for a total of 497 events: 430 events were mild, 61 were moderate, and 6 were severe. Overall, 41 patients (74.5%) had an AE related to disease progression (230 events), six patients (10.9%) had an AE related to idursulfase treatment (20 events), and five patients (9.1%) had an AE related to a study procedure (12 events). Serious AEs (SAEs) were reported in nine patients (16.4%; 13 events). There were no life-threatening AEs and no deaths during the study (Table 8). The most common AEs were pyrexia, upper respiratory tract infection, diarrhea, and carpal tunnel syndrome, reported in 14 (25.5%), 12 (21.8%), 10 (18.2%), and 10 (18.2%) of patients, respectively. The only SAE to occur in more than one patient was device-related infection (central venous access catheter), with three events (one mild and two moderate) seen in two patients (3.6%) and all resolved with antibiotic treatment (Table 8).

**Table 8:** Summary of study AEs and SAEs

AE description	Patients (N = 55), n (%)	Events, n
At least one AE	49 (89.1)	497
At least one AE related to disease progression	41 (74.5)	230
At least one AE related to treatment	6 (10.9)	20
At least one AE related to study procedures	5 (9.1)	12
At least one life-threatening AE	0	0
Deaths	0	0
Most common AEs (occurring in > 10% of patients)		
Pyrexia	14 (25.5)	20
Upper respiratory tract infection	12 (21.8)	19
Diarrhea	10 (18.2)	15
Carpal tunnel syndrome	10 (18.2)	12
Nasopharyngitis	9 (16.4)	16
Vomiting	9 (16.4)	12
Pain in extremity	8 (14.5)	8
Cough	7 (12.7)	10
Ear infection	7 (12.7)	10
Mitral valve incompetence	7 (12.7)	8
Aortic valve disease	7 (12.7)	7
Enlarged cerebral perivascular spaces	6 (10.9)	6
Otitis media	6 (10.9)	10
Most common SAEs (occurring in two or more patients)		
Device-related infection	2 (3.6)	3

AEs that occurred at or after informed consent till the EoS visit date plus 30 days are included

Proportions of patients are based on the total number of patients in the enrolled population

AEs are coded using the Medical Dictionary for Regulatory Activities (version 16.1)

AE, adverse event; EoS, end-of-study; SAE, serious adverse event

## Discussion

Obtaining a greater understanding of the natural history of cognitive function in patients with MPS II can help to inform the design of clinical trials of investigational therapies to treat the neurological manifestations of MPS II and contribute to better disease management. In this observational study, we found that cognitive ability as measured by the DAS-II varied widely among patients with MPS II, with a broad range of individual DAS-II GCA scores reported at baseline. Changes from baseline in DAS-II GCA scores also varied widely for individual patients.

Compared with the relatively early, rapid decline in cognitive impairment reported previously for some patients with neuronopathic MPS II [2], the natural course of cognitive impairment for some of the patients in this study was distinctly different, suggesting that certain patients may have some initial cognitive impairment, followed by a slow rate of deterioration after a period of relative stability. Indeed, we identified four possible subgroups of patients based on individual baseline DAS-II GCA scores and changes in these scores over time: patients who had marked early impairment followed by a rapid deterioration; patients who had marked early impairment followed by stabilization; patients with mild early impairment followed by stabilization; and patients whose scores suggested no initial impairment and remained stable. Various terminology (‘acute’, ‘chronic’, ‘pseudo-neuronopathic’, ‘severe’, ‘intermediate’, ‘attenuated’) could be used to describe the nature of the neuronopathy and cognitive impairment, but achieving consensus on the most appropriate terms is challenging. Of note, the pattern of decline in individuals with particularly severe cognitive impairment could not be ascertained because the study entry criteria (specifically the requirement of a DAS-II GCA score ≥ 55) excluded any such patients. Given that all patients enrolled received IV idursulfase, differences in somatic treatment are less likely to be a confounding factor. As with DAS-II GCA scores, adaptive behavior as measured by changes from baseline in VABS-II ABC scores varied considerably in individual patients during the study.

The finding that neurodevelopmental changes in individual patients with MPS II follow highly variable trajectories is perhaps to be expected given the variable progression of the disease. As outlined above, a binary classification of MPS II into neuronopathic and non-neuronopathic disease may be too simplistic, and there may be further patient subgroups that can be identified, as also postulated in a study of patients with MPS II in England [19]. This has also been seen in patients with other MPS disorders such as MPS I, in which cognitive impairment is heterogenous and varies depending on age at treatment (HSCT or ERT), genotype, and somatic disease burden [29].

Findings from a study (published after the current study was performed) also suggest the rate of cognitive decline in patients with MPS II may be influenced by genotype. Seo et al. evaluated change in cognitive ability (assessed using the Kyoto Scale of Psychological Development) in 13 Japanese boys aged ~ 4 to 10 years who had neuronopathic MPS II and were receiving IV ERT [30]. Patients were divided into two groups based on *IDS* variant type: those with missense variants, who were hypothesized to have a milder form of disease (mean follow-up of 44.0 months), and those with deletions, recombinations, and nonsense variants, who were hypothesized to have a more severe form of disease (mean follow-up of 38.3 months). During the study, the rate of cognitive decline was less rapid in patients with missense *IDS* variants than in those with non-missense *IDS* variants (mean change from baseline to last visit in developmental quotient, 29.4 vs. 31.2, respectively; *P* = 0.00033); however, whether the size of the difference between the two groups was clinically relevant remains to be elucidated [30].

Overall, in the present study, cognitive function as assessed by the DAS-II GCA score at baseline was low. There were modest changes from baseline in DAS-II GCA score and its component cluster scores over 24 months; small reductions in DAS-II GCA score at 24 months were observed in the subgroup of patients aged < 7 years at baseline or with a DAS-II GCA score ≤ 70 at baseline.

Adaptive behavior as measured by the VABS-II was moderately low at baseline; modest changes from baseline were observed in the VABS-II ABC standard scores and component domain scores over the 24 months. When baseline age was considered, the LSMD in the change from baseline in VABS-II ABC score between the two age subgroups (aged < 7 years and aged ≥ 7 years) was greater at month 24 than it was at month 12; it is possible that this difference would have continued to increase if patients had been followed up for more than 24 months.

There were no notable safety findings in this observational study. Most AEs were mild in severity and attributable to disease progression. There were few SAEs; none were life-threatening, and all resolved with treatment.

Given that this study included only a small subset of patients with MPS II, interpretation of these data is likely to be of limited use for understanding the changes in cognitive function and adaptive behavior that may occur in the broader MPS II population. The study population may not have been representative of the overall MPS II patient population because the study selection criteria dictated the exclusion of patients aged < 2 years and those with more severe cognitive impairment (DAS-II GCA score < 55). Indeed, a key limitation of the study was the requirement for patients to have a DAS-II GCA score ≥ 55, which resulted in the exclusion of both younger patients with early, severe cognitive impairment and older patients who may have experienced early severe cognitive impairment, but remained stable thereafter. Thus, the population may have been skewed towards patients with less severe cognitive impairment and whose cognitive function was relatively intact.

It is also important to highlight that our study was not a true natural history study for two reasons. Firstly, study entry criteria restricted patients with very low cognitive ability to be studied and secondly, patients were not required to remain in the study and could screen for and enroll into the phase 2/3 intrathecal enzyme replacement clinical trial [27]. An accurate natural history study would have required patients to complete 1 year of natural history before having the opportunity to screen for an investigational study. The loss of patients to the phase 2/3 study (as evidenced by the declining number of patients over time) is an important limitation that may have skewed the population further. This is because some of the inclusion criteria for the phase 2/3 study took into account the rate of cognitive decline of patients in the current study. At phase 2/3 trial screening, patients aged ≥ 3 and < 13 years were required to have either a DAS-II GCA score of 55–85, or a DAS-II GCA score > 85 plus evidence of a ≥ 10-point decrease in DAS-II GCA score over 12 months in the current study. Patients aged ≥ 13 and < 18 years were required to have both a DAS-II GCA score of 55–85 at phase 2/3 trial screening and evidence of a ≥ 10-point decrease in DAS-II GCA score over 12 months in the current study [27]. It is clear that the overall characteristics of those patients who discontinued early were different from those of patients who remained on study. The patients leaving the study early were notably younger and a greater proportion had a severe disease phenotype. The cumulative loss of data from patients who discontinued to enroll into the phase 2/3 trial (especially given that most participants who discontinued the study did so during the first study year) may well have resulted in an apparent stabilization of overall mean DAS-II GCA scores as the study population became enriched with patients who, relative to the full baseline population, had only mild MPS II and a reduced propensity for further changes in cognitive function. Conversely, it is also possible that a few of the patients who remained in this study may have been those with cognitive impairment too severe to qualify for the phase 2/3 trial (i.e. their DAS-II GCA score had fallen below 55, following entry into this current study). This would have led to the population in this current study becoming enriched with patients who had severe cognitive impairment and a propensity for rapid worsening in cognitive function, potentially increasing the variation in the data. Therefore, taken together, the data from this study may not be a true representation of the full spectrum of natural disease course in patients with MPS II. Nonetheless, the results provide useful information on the variability in trajectories of cognitive function decline in patients with MPS II.

There are several challenges associated with cognitive testing in patients with MPS II that may have contributed to the variability of our results. For example, DAS-II scores for individual patients varied considerably between visits (e.g. dropping by over 20 points between two visits and then returning to the original, higher score at the next visit). This may have been driven by the children having ‘good days’ and ‘bad days’, which may have been caused by factors such as poor sleep, medical complications, major schedule changes, or novel circumstances [12]. Indeed, the results of a subgroup analysis of 38 children from this study with below average–very low abilities (DAS-II GCA scores of 55–85 at any time during the study) suggest that age-specific clinical trial endpoints may be needed when assessing cognitive ability [31].

We should also acknowledge that the DAS-II is expressed as norm-based scores. These are limited in their ability to assess absolute change in cognitive ability between timepoints in children such as those in this study, whose cognitive development may progress, but more slowly than in healthy same-age peers. The Projected Retained Ability Score has been proposed as an approach to use norm-based scores to characterize absolute change over time and overcome this issue [32]. However, in 2013, when enrollment into our study started, the DAS-II was considered an appropriate assessment. Since then, experts have convened to provide recommendations on the cognitive endpoints for therapy development for neuronopathic mucopolysaccharidoses, first in 2016 [33] and again in 2020 [34]. The earlier consensus recognized the utility of the DAS-II and described it as an attractive option for longitudinal studies within English- or Spanish-speaking populations [33]. However, for children over the age of 3 years, as in our study, the Wechsler scales have been recommended more recently to measure cognitive outcome in patients with less severe cognitive impairment, with a nonverbal scale such as the Kaufman Assessment Battery for Children, Second Edition Non-Verbal Index recommended for patients who find the Wechsler tests too difficult to perform [34].

In particular, the frequency of cognitive testing in this study (every 3 months) may have been too often and could have affected the results. Boys may have become frustrated, irritated, or bored by the regularity of the tests and less willing to co-operate. Additionally, overly frequent cognitive testing can have a learning or practice effect, although this may have less of an impact in patients with MPS II. In addition, when age-equivalent scores were examined, ceiling and floor scores were observed and the statistical descriptions of age-equivalent scores were not meaningful (data not shown). Physical manifestations of the disease, such as hearing difficulties and impaired motor function, can also affect some components of the DAS-II assessment [35, 36]. Additionally, although behavioral issues associated with MPS II can impact the testing of cognitive functioning, and can make it difficult to perform the tests, experienced examiners often employ various strategies to optimize the testing experience [36, 37]. These include allowing the child to play intermittently during testing, or allowing extra time if the patient has a slower response; examiners are advised to adhere to the requirements of the test, but be flexible where necessary based on the needs of the child [36]. A final potential limitation of the study was its duration: it is likely that a period of more than 2 years is required to assess changes in cognitive ability effectively in patients with MPS II, owing to the variability and progressive nature of the disease, the characteristics of the patient population, and the challenges associated with performing and interpreting cognitive/behavioral tests discussed above.

There are several learnings from this study that may inform the design and analysis of future studies for rare, progressive diseases such as MPS II. For example, the timing of cognitive assessments should be carefully considered to ensure that these are not scheduled too frequently. It may have been more appropriate to test cognitive function and adaptive behavior every 6 months rather than every 3 months. For future studies, it will be important to define the population of patients who may benefit most from a therapy to treat the neurological manifestations of MPS II and to be able to measure the effects of any such treatments. It is likely that earlier intervention would be more effective than later intervention (as seen in patients with MPS I treated with HSCT [38]); thus, it will be important to define early in their disease course the subgroup(s) of patients with MPS II who may be expected to experience cognitive function decline in the short-to-medium term.

## Conclusions

In this study, some patients with MPS II experienced rapid declines in cognitive ability, whereas others remained stable, albeit impaired, cognitive function over time. The greatest changes in DAS-II GCA score at 24 months were observed in subgroups of patients aged < 7 years at baseline or who had a DAS-II GCA score ≤ 70 at baseline. The heterogeneity of the study population with respect to disease severity and the extent of neurological involvement may have limited interpretation of the overall results, given that cognitive function and adaptive behavior appeared to remain relatively stable over 24 months for the overall patient population. These initial insights provide a basis for more detailed future comparative analyses between patient subgroups, which may enhance the definition and understanding of factors that influence cognitive and adaptive function in MPS II.

## Data Availability

The datasets, including redacted study protocol, redacted statistical analysis plan, and individual participants’ data supporting the results reported in this article, will be made available within 3 months from initial request, to researchers who provide a methodologically sound proposal. The data will be provided after de-identification in compliance with applicable privacy laws, data protection, and requirements for consent and anonymization.
